# Oxidative Stress-Related Transcription Factors in the Regulation of Secondary Metabolism

**DOI:** 10.3390/toxins5040683

**Published:** 2013-04-18

**Authors:** Sung-Yong Hong, Ludmila V. Roze, John E. Linz

**Affiliations:** 1 Department of Food Science and Human Nutrition, Michigan State University, East Lansing, MI 48824, USA; E-Mails: hongsun7@msu.edu (S.-Y.H.); roze@msu.edu (L.V.R.); 2 Department of Microbiology and Molecular Genetics, Michigan State University, East Lansing, MI 48824, USA; 3 National Food Safety and Toxicology Center, Michigan State University, East Lansing, MI 48824, USA; 4 Center for Integrative Toxicology, Michigan State University, East Lansing, MI 48824, USA

**Keywords:** aflatoxin, aspergilli, oxidative stress, secondary metabolism, stress activated signaling pathway, transcription factor

## Abstract

There is extensive and unequivocal evidence that secondary metabolism in filamentous fungi and plants is associated with oxidative stress. In support of this idea, transcription factors related to oxidative stress response in yeast, plants, and fungi have been shown to participate in controlling secondary metabolism. Aflatoxin biosynthesis, one model of secondary metabolism, has been demonstrated to be triggered and intensified by reactive oxygen species buildup. An oxidative stress-related bZIP transcription factor AtfB is a key player in coordinate expression of antioxidant genes and genes involved in aflatoxin biosynthesis. Recent findings from our laboratory provide strong support for a regulatory network comprised of at least four transcription factors that bind in a highly coordinated and timely manner to promoters of the target genes and regulate their expression. In this review, we will focus on transcription factors involved in co-regulation of aflatoxin biosynthesis with oxidative stress response in aspergilli, and we will discuss the relationship of known oxidative stress-associated transcription factors and secondary metabolism in other organisms. We will also talk about transcription factors that are involved in oxidative stress response, but have not yet been demonstrated to be affiliated with secondary metabolism. The data support the notion that secondary metabolism provides a secondary line of defense in cellular response to oxidative stress.

## 1. Introduction

Living organisms including fungi and plants use a variety of signal transduction mechanisms to sense and respond to different forms of environmental stress. This review is focused on those signaling pathways, which are activated in response to reactive oxygen species (ROS) such as hydrogen peroxide and superoxide anion. The classical view of the response to oxidative stress was developed based on studies in yeast which demonstrated that modulation of transcription of defense-related antioxidant genes assists in the survival of the organism. Like yeast, filamentous fungi must respond to oxidative stress, but due to the variety of environmental conditions with which they cope, their response is more robust and complicated than that of yeast. One factor in particular contributes to the complexity of filamentous fungal response to oxidative stress. Recent studies provide solid support for the notion that regulation of secondary metabolism is closely linked to the cellular response to oxidative stress in filamentous fungi [1,2,3,4,5]. The available data strongly suggest that several transcription factors associated with the stress activated protein kinase/mitogen-activated protein kinase (SAPK/MAPK) pathway coordinate the timing and level of expression of target genes including antioxidant and secondary metabolism genes, thus controlling metabolic processes with cellular stress response. In aspergilli*,* it was proposed that antioxidant enzymes represent the first line of defense against excessive ROS formation and that synthesis of secondary metabolites functions as a second line of defense from ROS damages [1,5]. 

In this review, we focus on oxidative stress-related transcription factors, which have been shown to contribute in co-regulation of secondary metabolism, in particular aflatoxin biosynthesis, with oxidative stress response in the aspergilli. Several of these transcription factors are directly coupled to the SAPK/MAPK pathway. In addition, based on functional analyses of transcription factors, such as the CCAAT binding complex and Myb transcription factors, we make several predictions for the involvement of specific transcription factors, not yet reported, in co-regulation of aflatoxin biosynthesis with oxidative stress. 

## 2. Stress Activated Signaling Pathways in Yeast and Filamentous Fungi

**Figure 1:**
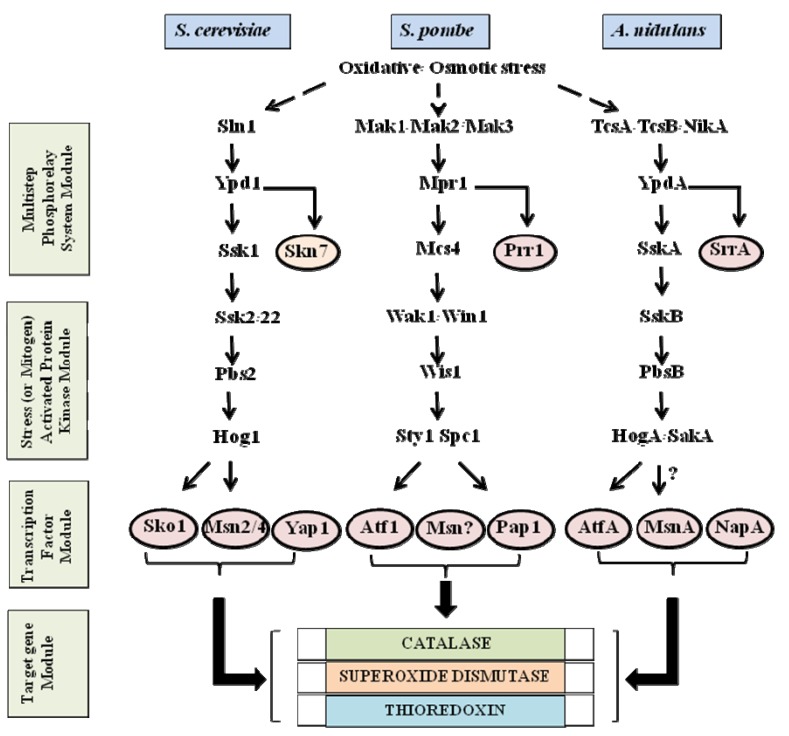
Stress activated signaling pathways in *Saccharomyces cerevisiae*, *S. pombe*, and *Aspergillus nidulans*. In *S. pombe* and *A. nidulans*, Sty1/Spc1 (HogA/SakA) is activated by environmental stress such as oxidative and osmotic stress and induces transcription factors such as Atf1 (AtfA) for target gene expression. On the other hand, Hog1 activation for Sko1 in *S. cerevisiae* is dependent on osmotic stress. The TcsA/TcsB/NikA sensor kinases transmit oxidative stress signals to the HogA/SakA SAPK/MAPK cascade through YpdA and SskA in *A. nidulans* while Sln1 sensor kinase transmits osmotic stress signals to the Hog1 SAPK/MAPK cascade through Ypd1 and Ssk1 in *S. cerevisiae*. The Skn7 response regulator is also under the Sln1-Ypd1 signal transduction, but oxidative stress appears to activate Skn7 independently of the phosphorelay system. The solid lined arrows indicate signal transduction between two proteins and the dotted arrows indicate signal transduction from oxidative and/or osmotic stress. The thick lined arrows designate binding of transcription factors to target gene promoters. The circles around the proteins indicate transcription factors. Unknown signal transduction between two proteins and unidentified transcription factors are shown by question marks.

The signaling pathways that participate in response to a number of stresses (osmotic, UV irradiation, high temperature) including oxidative stress were comprehensively dissected in yeast and were found to be evolutionally conserved in filamentous fungi. These conserved signaling pathways incorporate a multistep phosphorelay system module (an analog of the bacterial two-component system), which relays the signal to the stress activated protein kinase/mitogen-activated protein kinase (SAPK/MAPK) pathway module, and this modulates activity of an array of specific oxidative/osmotic stress related transcription factors (Figure 1) [6,7,8,9,10]. The transcription factors, depending on their activation state, alter expression of the target genes involved in the cellular response to the stress signals. The components of these signaling cascades and the mechanisms of the signal transduction have been extensively explored and documented [6,8,9,10,11,12]. 

The essential elements of the multistep phosphorelay system module include sensor hybrid histidine kinases (Sln1/Mak/Tcs/NikA), histidine-containing phosphotransfer intermediates (Ypd1/Mpr1/YpdA), and response regulators (Ssk1/Mcs4/SskA) [6,13]; these signaling elements convey the phospho-signal from a conserved histidine to an aspartate residue thus relaying the signal to the SAPK/MAPK module. The SAPK/MAPK signaling module is highly conserved between yeast, filamentous fungi, and higher eukaryotes [8,14,15,16,17]. The module consists of a major MAPK (Mitogen-Activated Protein Kinase), which phosphorylates target transcription factors and other cytosolic proteins on serine and threonine residues within a consensus motif. MAPK is activated via dual phosphorylation of conserved threonine and tyrosine residues, located in the activation loop, by MAPK kinase (MAPKK, MEK). MAPKK, in turn, is activated by MAPKK kinase (MAPKKK, MEKK).

The bZIP transcription factors that belong to the CREB/ATF (cAMP response element binding protein/activating transcription factor) family are highly conserved between yeast and filamentous fungi in structure (a basic motif for DNA binding and a leucine-zipper motif for dimerization) and regulatory mechanisms. bZIPs are controlled not only by MAPK, but also by cAMP-PKA (Protein Kinase A) activity. Atf1 and Pap1 are homologous to human ATF2 and c-Jun, respectively. Most target genes of these transcription factors are antioxidant-related genes such as catalase, thioredoxin, and superoxide dismutase which mediate cellular defense against oxidative stress [18]. 

A ROS signal can be incorporated into the signaling pathway at different levels. For example, extra- and intra-cellular ROS can be perceived by the membranous and cytosolic sensor histidine kinases; it has been suggested that PAS/PAC and GAF domains adjacent to the histidine kinase domain, are likely involved in ROS sensing [8,19]. In *Schizosaccharomyces pombe*, an oxidative stress signal can be transmitted to the SAPK/MAPK module independently of the phosphorelay system module suggesting a direct ROS activation mechanism on Wak1/Win1 MEKK or involvement of a novel zinc-finger transcription factor Hsr1 signaling circuit [20,21]. Several transcription factors such as AP-1 homologs and CCAAT-binding transcription factors (see below) can be directly engaged in signaling by redox stimulus [7,22] or by modulation of AP-1 activity with the involvement of the thioredoxin system [23,24]. This highly conserved regulatory network that includes signaling modules, transcription factors, and target genes provides a robust defense system against oxidative stress in yeast and filamentous fungi. 

Despite conservation of the major signaling modules, filamentous fungi, in contrast to yeast, have additional mechanisms to cope with ROS, such as the presence of a larger number of sensor histidine kinases [10], antioxidant enzymes, and production of secondary metabolites with antioxidant function [12]. In *Aspergillus parasiticus*, it has been shown that there is a correlation between ROS formation, activation of oxidative stress-related transcription factors (ApyapA, AtfB, and MsnA), antioxidant enzyme activation, and production of the secondary metabolite aflatoxin, suggesting that oxidative stress triggers aflatoxin production [1,3,5,25,26]. Several oxidative stress-related transcription factors (Apapy1, AtfB, Nap1) associated with the SAPK/MAPK signaling cascade, regulate secondary metabolism in aspergilli [5,25,27]. These data indicate that incorporation of secondary metabolism into oxidative stress response occurs at least in part at the level of the transcription factors. Therefore, transcription factors (transcription factor module) are of special interest as they define metabolic decisions during stress response.

## 3. Transcription Factors Related to Oxidative Stress, Which Are Involved in Regulation of Secondary Metabolism

Existence of transcription factors with dual functionality (toward ROS signaling and secondary metabolism) serves as a strong support to the statement that secondary metabolism is a crucial part of the cellular response to oxidative stress in filamentous fungi; these transcription factors are the primary focus of this chapter. We will also present an interesting example of a transcriptional regulator, CCTTA transcription factor complex, which possesses a set of characteristics that provide the complex with the potential to serve as a co-regulator of aflatoxin biosynthesis and oxidative stress.

### 3.1. AP-1

AP-1 (Activating Protein 1), a bZIP transcription factor in yeast and filamentous fungi, is a transcriptional activator expressed in response to oxidative stress [9,25,28]. AP-1 is one of the immediate targets for regulation by SAPK/MAPK. AP-1 binds to an AP-1 binding site (5'TGAC/GTCA3', 5'TT/GACTAA3'), which is highly similar to CRE (5'TG/TACGTC/AA3'), in the promoters of target genes and carries out multiple functions as a red-ox regulator [29,30]. 

Fungal AP-1 transcription factors contain *N*-terminal and *C*-terminal cysteine rich domains [7]. The AP-1 activation mechanism is well known for the yeast Yap1. Upon exposure to H_2_O_2_, 2 or more cysteines in the *C*-terminal cysteine rich domain (c-CRD) of Yap1 undergo direct oxidation and form intramolecular disulfide bond [31]. The oxidized form of Yap1 is transcriptionally active and is retained in the nucleus due to conformational changes that prevent its interaction with the export receptor Crm1/Xpo1 and removal from the nucleus. Yap1 deactivation involves enzymatic reduction of the oxidized form of Yap1 by thioredoxin whose transcription is increased by activation of Yap1 [7]. 

The yeast AP-1 family of transcription factors includes Yap1 of *Saccharomyces cerevisiae*, Pap1 of *S. pombe*, Cap1 of *Candida albicans*, and Kap1 of *Kluyveromyces lactis* [7]. These homologs regulate the expression of a number of genes involved in oxidative stress response, including *CTT1* (cytosolic catalase), *TRX2* (thioredoxin), *TRR1* (thioredoxin reductase), *SOD1* (Cu/Zn superoxide dismutase), *TSA1* (thioredoxin peroxidase), and *GLR1* (glutathione reductase) [7,32]. It was reported that Yap1 binds to the Yap1 response element (5'TT/GAC/GT/AAA3'; YRE) in target genes [30,33,34]. Similarly, Toda and co-workers reported that Pap1 binds to the sequence 5'TTAGTCA3' in target genes [35]. It is also known that Yap1-dependent transcriptional activity is inhibited by cAMP-dependent PKA [30]. 

In aspergilli, Yap1 orthologs were identified in *A. nidulans*, *A. fumigatus*, and *A. parasiticus*. In *A. nidulans*, NapA (a Yap1 ortholog) was reported to play an important role in cellular defense against oxidative stress such as hydrogen peroxide and superoxide radicals [36]. NapA was shown to be a transcriptional activator of oxidative stress associated genes such as *catB* (mycelia-specific catalase), *trxB* (thioredoxin reductase), *thiO* (thioredoxin), and *glrA* (glutathione reductase) in response to hydrogen peroxide [36]. Of particular importance, NapA functioned coordinately with SrrA and/or SskA response regulators in response to oxidative stress in similar fashion as Yap1 cooperates with Skn7 in yeast [34,37]. In addition, overexpression of *napA* resulted in an increased tolerance to oxidative stress and decreased secondary metabolite production in *A. nidulans*, including sterigmatocystin, emericellin, asperthecin, shamixanthone, and epishamixanthone [27]. 

Deletion of Ap*yapA* (*A. parasiticus* ortholog of *YAP1*) in *A. parasiticus* resulted in an increased susceptibility to extracellular oxidants, precocious ROS and aflatoxin accumulation, and premature conidia formation as compared to the wild type strain [25]. In addition, the Ap*yapA* disruptant produced more hydroperoxides and aflatoxin in maize seeds compared to the wild type strain, suggesting a correlation between oxidative stress and aflatoxin biosynthesis [26].

Similar to the Ap*yapA* disruptant, deletion of Ao*yap1* in *A. ochraceus* resulted in increased ochratoxin production and higher amounts of ROS formation compared to the wild type strain [2]. In addition, expression of *catA* (conidia-specific catalase) and Cu, Zn *sod1* (Cu, Zn-superoxide dismutase) in the Ao*yap1* deletion mutant was down-regulated.

### 3.2. AtfA

The bZIP transcription factor AtfA in *A. nidulans* regulates oxidative and osmotic stress responses and activates expression of *catB* by binding to CRE sites in its promoter in response to H_2_O_2_ [38]. In addition to being regulated by SAPK/MAPK, AtfA is also controlled by cAMP/PKA. It was reported that AtfA plays a critical role in regulation of *catA* during fungal development [39]. AtfA was also shown to play an important role in tolerance of conidia to oxidative stress via interaction with SakA while it played a minor role in a mycelium resistance to H_2_O_2_ [39,40]. Interestingly, Lara-Rojas and co-workers suggested that AtfA and AtfB may interact to regulate some genes [39]. Considering the interaction of AtfA and AtfB in oxidative stress response, it is reasonable to anticipate that AtfA may have a possible connection with secondary metabolism. 

In a plant pathogen *Botrytis cinerea*, an *A. nidulans* AtfA ortholog BcAtf1 is partially targeted by SAPK cascade signaling and is induced at transcription level upon exposure to H_2_O_2_ independently of SAPK [41]. Similar to *A. nidulans* Atf1, BcAtf1 controls expression of catalase B but is not involved in overall tolerance to osmotic and oxidative stress as measured by colony growth. Interestingly, *bcatf1* deletion mutant accumulates 2 to 10 fold higher levels of secondary metabolites (polyketide botcininA, sesquiterpene botrydial, and botryendial) [41]. Accumulation of phytotoxins correlated with up-regulation of expression of genes involved in toxin biosynthesis suggesting that BcAtf1functions as a repressor of genes involved in biosynthesis of secondary metabolites in *B. cinerea*. Although a yeast one-hybrid analysis did not show a direct interaction of BcAtf1 with the promoters of biosynthetic genes, the data strongly demonstrate indirect influence of BcAtf1 on gene expression likely through interaction with other regulators (see above and below). 

In *S. pombe*, Atf1 (an ortholog of AtfA in *A. nidulans*) is activated via phosphorylation by Sty1 in the SAPK signaling cascade. Atf1 induces expression of *ctt1* (cytosolic catalase), *gpx1* (glutathione peroxidase), and *trr1* (thioredoxin reductase) in response to oxidative stress [7,8,20]. Atf1 was shown to activate the transcription of some target genes by forming a heterodimer with a small bZIP transcription factor Pcr1, and Atf1 and Pcr1 have similar roles in response to oxidative stress [42,43]. In contrast to *A. nidulans* and *S. pombe,* Sko1 (an ortholog of Atf1 in *S. pombe*) in *S.* *cerevisiae* binds to CRE sites in the promoters of target genes and acts as a transcriptional repressor under normal growth conditions but induces expression of target genes after activation by phosphorylation in response to osmotic stress [44]. In this regard, filamentous fungi are closer to fission yeast than to budding yeast. 

### 3.3. AtfB

**Figure 2:**
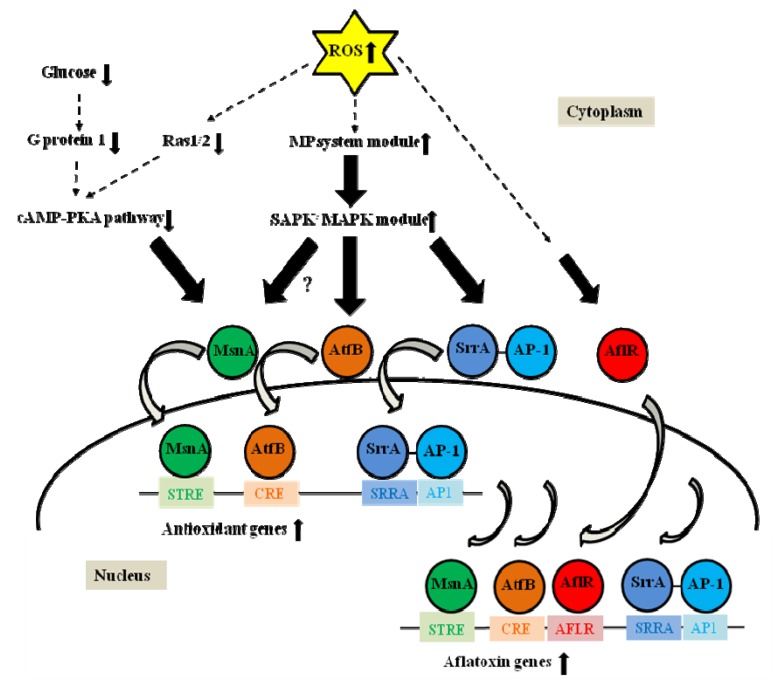
A model for transcriptional activation of antioxidant and aflatoxin biosynthetic genes by oxidative stress-related transcription factors. Based on available experimental evidence, the model proposes that increased levels of intracellular reactive oxygen species (ROS) in fungal cells down-regulate the cAMP-PKA signaling pathway. This promotes MsnA binding to STRE sites in promoters of antioxidant genes for their activation. Simultaneously, ROS up-regulate the stress activated protein kinase/mitogen activated protein kinase (SAPK/MAPK) signaling cascades through the multistep phosphorelay system. Activation of the SAPK/MAPK cascade promotes AtfB and SrrA binding (SrrA recruits AP-1) to corresponding CRE, SRRA, and AP1 sites in promoters of the antioxidant genes for their induction. Then MsnA, AtfB, and SrrA bind (SrrA recruits AP-1) to corresponding STRE, CRE, SRRA, and AP1 sites in promoters of aflatoxin genes for their activation due to excessive levels of ROS. AflR assists in induction of aflatoxin biosynthetic genes by binding to AFLR sites in the aflatoxin gene promoters. The dotted arrows indicate signal transduction from ROS and the solid lined arrows indicate signal transduction pathways. The curved arrows designate entering of transcription factors from cytoplasm to nucleus and binding of the transcription factors to the corresponding recognition motifs. Undefined yet signal transduction between MsnA and SAPK/MAPK module is shown by a question mark. PKA, protein kinase A; MP, multistep phosphorelay; SAPK/MAPK, stress activated protein kinase/mitogen activated protein kinase.

AtfB is a member of the CREB/ATF family that binds CRE sites (5'TG/TACGTC/AA3'). An *atfB* disruptant of *Aspergillus oryzae* showed decreased transcription of antioxidant target genes such as *catA* (conidia-specific catalase) and produced conidia sensitive to H_2_O_2_ [45]. *atfB* was expressed in the late growth phase during conidiation on solid media. In *A. parasiticus*, AtfB was shown to bind to promoters of aflatoxin biosynthetic genes including *nor-1*, *fas-1*, *ver-1*, and *omtA*, which carry CRE sites [3,5]. Mutations in the CRE binding site in the *nor-1* promoter reduced *nor-1* transcription upon induction by exogenous cAMP. These data indicated that cAMP/PKA pathway regulates aflatoxin biosynthesis at least in part through AtfB [46]. Expression of *atfB* correlated with aflatoxin gene expression under aflatoxin-inducing conditions, suggesting that AtfB activates the aflatoxin gene promoters carrying CRE sites. AtfB has been demonstrated to be an important co-regulator that binds to the promoters of many aflatoxin genes including *fas-1* and *ver-1* as well as stress response genes such as mycelia-specific *cat1* and mitochondria-specific Mn *sod* [5]. Thus, AtfB regulates coordinate expression of the aflatoxin genes and antioxidant genes. Each of the promoters that bind AtfB carries CRE sites whereas the *vbs* promoter (an aflatoxin gene), which lacks a CRE site, does not bind AtfB [46]. Recent data also support the existence of a transcription factor regulatory network that consists of AtfB, AP-1, MsnA, SrrA, and AflR, and this network, together with other signaling components, promotes fungal response to oxidative stress [5,46]. We proposed that increased levels of intracellular ROS activate antioxidant and aflatoxin genes via two signal transduction pathways. First, ROS down-regulate the cAMP-PKA pathway, which results in MsnA binding to the target promoter and activation of the antioxidant gene*.* Simultaneously, ROS up-regulate the SAPK/MAPK signaling cascade through the multistep phosphorelay system, which results in AtfB and SrrA binding to the promoters and induction of the antioxidant genes. MsnA, AtfB, and SrrA (SrrA recruits AP-1) then bind to the promoters of aflatoxin biosynthetic genes to assist in their induction by zinc binuclear cluster (C6) transcription factor AflR (Figure 2) [5].

### 3.4. MsnA

MsnA2 and MsnA4 in *S. cerevisiae* are Cys2His2 zinc finger transcription factors that are induced as part of the cellular response to oxidative stress as well as other types of stresses such as carbon starvation, heat shock, and osmotic stress [47]. MsnA2 and MsnA4 activate transcription of stress response genes such as *CTT1* (catalase T) and *HSP12* (heat shock protein 12) by binding to the stress response element (5'AGGGG3') in their promoters. It is known that Msn2/4 activity is inhibited by cAMP-dependent protein kinase (PKA).

Deletion of *msnA* (an ortholog of *MSNA2*) in *A. parasiticus* and in *A. flavus* resulted in growth inhibition but increased production of conidia, ROS, aflatoxin, and kojic acid [48]. *msnA* disruption up-regulated expression of genes encoding enzymes that protect against ROS (e.g., superoxide dismutase, putative catalase, and cytochrome c peroxidase in *A. parasiticus* and catalase A in *A. flavus*). These findings may imply that deletion of *msnA* in *A. parasiticus* and in *A. flavus* down-regulates expression of mycelial catalase (in *A. parasiticus*) and of catalase B (in *A. flavus*) which in turn up-regulates expression of other antioxidant genes including the putative catalase and superoxide dismutase for fungal defense to compensate for the decreased expression of the mycelial catalase and catalase B and to cope with higher levels of ROS. 

### 3.5. StuA

Incomplete data exist on the helix-loop-helix developmental regulator StuA in regard to its involvement in co-regulation of secondary metabolism and oxidative stress response. Expression of the *cpeA*, a gene that encodes for a Hülle cell-specific bifunctional enzyme catalase-peroxidase, is under positive influence of StuA in *A. nidulans* [49]. On the other hand, in *A. fumigatus*, six secondary metabolic gene clusters were found to be under control of StuA [50]. 

### 3.6. CCAAT-Binding Transcription Factor Complex

Eukaryotes including fungi, plants, and mammals possess evolutionally conserved orthologs of a multi-subunit Hap (Heme Activator Protein) complex consisting of at least three subunits in filamentous fungi, plants, and vertebrates, and four subunits in yeast [51,52,53,54,55]. The complex binds specifically to a CCAAT motif within promoters of target genes involved in primary metabolism, cell-cycle, development, stress response, and particularly oxidative stress, and controls expression of the target genes (see below). In plants and vertebrates, each histone-like component of the heterotrimeric complex (known as NF-Y, Nuclear Factor Y, or CBF, CCAAT-binding factor) is required for subunit interactions, CCAAT binding through histone fold motifs, and bifunctional regulation of the target gene expression [53,54,55]. In mammalian cells, it has been shown that NF-Y affects the histone acetylation landscape in gene promoters, thus influencing gene transcription [56]. The Hap complex binding motif CCAAT was first identified in yeast [57,58]. The yeast Hap complex plays a role as a transcriptional regulator during respiration and oxidative stress response and induces genes such as *CYC1* (cytochrome c-iso-1), *CYC7* (cytochrome c iso-2), *CYT1* (cytochrome c1), and *CTT1* (catalase). The heterotrimer consisting of Hap2p, Hap3p, and Hap5p, is essential for DNA binding, whereas a fourth component, Hap4p, carries a transcription activation domain. 

In the filamentous fungus *Aspergillus nidulans*, at least HapC, HapE, and HapB are required for assembly of a heterotrimeric complex called AnCF/PenR1/AnCP which is able to bind the consensus CCAAT motif and regulate gene transcription [51,59,60]. HapB, the only subunit that carries a nuclear localization signal sequence, is responsible for the translocation of the entire heterotrimeric complex to the nucleus [61,62].

The *A. nidulans* Hap-like complex(s) AnCF/PenR1 has been reported to control expression of genes involved in penicillin biosynthesis including *ipnA*, *acvA*, *aatA*, and *aatB* [63,64,65]. The CCAAT binding complex AnCF (*A. nidulans* CCAAT binding factor) exerts a positive influence on *ipnA*, *aatA*, and *aatB* transcription, whereas a negative effect on transcription of *acvA* was documented. A basic-region helix-loop-helix transcription factor AnBH1 has been found to bind to an asymmetric E-box 5'-AATCACAGG-3' which partially overlaps the CCAAT motif within the *aatA* promoter; these findings suggest competitive interactions between these transcription factors for binding to the promoter [66].

AnCF serves as a necessary signaling component in the cellular response to oxidative stress [22]. In particular, AnCF fulfills the role of a redox sensor in the cell. Three cysteine residues are located in conserved positions within the histone fold motif in all HapC subunits from fungi to humans; only a form of HapC that possesses all 3 cysteines in a reduced state, is able to assemble into a functional heterotrimeric AnCF complex. Although there currently is no direct indication for the involvement of an AnCF complex in the SAPK/MAPK pathway, the AnCF complex may regulate oxidative stress response indirectly in concert with NapA (*A. nidulans* ortholog of *S. cereviseae* Yap1), which is regulated by SAPK/MAPK [22]. Specifically, AnCF binds the consensus binding motif in promoters of *napA* and NapA target genes such as *catB* (mycelial catalase B), *trxA* (thioredoxin), and *prxA* (thioredoxin-dependent peroxidase), thus maintaining glutathione homeostasis [22].

In *Arabidopsis thaliana*, over-expression of one of the subunits of the NF-Y complex, NFYA5, affected expression of 130 genes including a number of genes involved in oxidative stress response, e.g., a subunit of cytochrome b6-f complex, glutathione *S*-transferase, peroxidases, and an oxidoreductase family protein [67]. However, no connection between the *A. thaliana* NF-Y complex and secondary metabolism was reported yet.

In *A. parasiticus*, a CCAAT motif was identified in promoters of many genes involved in aflatoxin biosynthesis, except for *aflR* (a pathway specific regulator) and *ver-1* (encoding a middle enzyme in the aflatoxin biosynthetic pathway) [46]. The intergenic region between the divergently transcribed *fas1* and *fas2* genes (encoding enzymes that catalyze hexanoate formation during the first step in aflatoxin biosynthesis) does not possess an AflR recognition site. Thus, it is not clear how *Aspergillus* coordinates *fas* gene transcription with the transcription of the other genes in the cluster. While missing the AflR recognition site, the *fas1/fas2* intergenic region does possess a Hap-like transcription complex binding site CCAAT and AtfB binding motifs [46]. Hap complex and AtfB represent strong candidates for playing a role in the mechanism that initiates transcription of the *fas1* and *fas2* genes and that coordinates their expression with transcription of other genes in the aflatoxin cluster as a part of the response to oxidative stress.

In other fungal species, CCAAT-binding complexes have been shown to regulate a number of genes encoding polysaccharide degrading enzymes such as Taka-amylase A (*taaG2*) in *A. oryzae* [60], xylan- and cellulose-degrading enzymes in *Trichoderma reesei* [68,69], and glutamate dehydrogenase in *Neurospora crassa* [70]. In *A. fumigatus*, a mutation in the CCAAT-binding transcription factor complex subunit HapE underlies a mechanism of resistance to azole, the primary antifungal agent for patients with mycoses caused by *A. fumigatu*s [71]. Although the CCAAT-binding complex in these fungi has not been shown to participate in secondary metabolism or oxidative stress response regulation or to be associated with SAPK/MAPK signaling pathway, the complex represents a strong extrapolative element in the regulatory network.

## 4. Transcription Factors with Strong Potential to Coordinate Oxidative Stress and Secondary Metabolism

A complexity of the transcription factor regulatory network that coordinates secondary metabolism with oxidative stress response has just begun to unravel. In this chapter we focus on a group of transcription factors that have a strong potential for the role in this network despite currently accessible data are incomplete. A number of transcription factors have been shown to regulate response to oxidative stress such as Hsf1, SrrA/Skn7/Prr1, Pcr1, and Myb. These transcription factors may play a direct or indirect regulatory role in regulation of secondary metabolism and as transcriptional coordinators although the validating data are not immediately available. For example, the transcription factor SrrA (an *Aspergillus* ortholog of yeast Skn7/Prr1) that has a prominent function in oxidative stress response in aspergilli and yeast, has been suggested as a regulator in aflatoxin biosynthesis [5] (see below). 

Undoubtedly, one of the greatest challenges of fungal biology is to understand the entire regulatory network that coordinates secondary metabolism and cellular response to oxidative stress. Making the entire picture even more intricate, the essential elements of this network must comprise a heterotrimeric complex VeA-LaeA-VelB, the light-responsive global regulator of secondary metabolism. The Velvet complex proteins are not transcription factors but function as global regulators of many aspects of fungal biology including secondary metabolism such as aflatoxin gene activation. Involvement of VeA in cellular response to oxidative stress has now begun to be elucidated. In ongoing studies, VeA has been shown to be involved in transcriptional activation of genes involved in antioxidant response, *cat1* (mycelial catalase) and *trxA* (thioredoxin). 

### 4.1. Hsf1

In *S. cerevisiae* Hsf1 (heat shock factor 1) binds as a homotrimer to the heat shock element (HSE) found in the promoters of the heat shock genes, the genes which are expressed under heat shock conditions causing damage of proteins such as denaturation. The element consists of tandem inverted repeats of the sequence 5'AGAAN3' (where N is any nucleotide) [72]. The Hsf1 undergoes phosphorylation, which activates transcription of *CUP1* (copper metallothionein) for cellular protection in response to oxidative stress, in particular superoxide anion [73]. In the presence of hydrogen peroxide, Hsf1 cooperates with Skn7 to induce heat shock gene expression [74]. Activation of Hsf1 in response to superoxide anion is inhibited by cAMP-dependent PKA activity [75]. It seems that heat shock gene activation via Hsf1 is involved in refolding the damaged proteins during recovery from severe oxidative stress [75]. Considering the involvement of Hsf1 in oxidative stress response and crosstalk between transcription factors in the transcriptional network such as cooperation of Hsf1 with Skn7, it is reasonable to anticipate that Hsf1 may have a possible connection with secondary metabolism. 

### 4.2. SrrA

*A. nidulans* SrrA (an ortholog of *S. cerevisiae* Skn7 and *S. pombe* Prr1) is a member of the multicomponent histidine-to-aspartate phosphorelay system (analogous to the two-component phosphorelay system in prokaryotes) in a signal transduction pathway that mediates cellular response to environmental stimuli [76,77]. This response regulator is a transcription factor that contains a mammalian heat shock factor (HSF)-like DNA binding domain adjacent to a receiver domain, which is essential for phosphorelay function [76,78]. Disruption of *srrA* in *A. nidulans* showed hypersensitivity to oxidative stresses such as hydrogen peroxide and decreased levels of expression of mycelia-specific *catB* (catalase B) [76,77]. 

Skn7 in *S. cerevisiae* is a winged helix-turn-helix transcription factor that has been shown to physically interact with either Yap1 (an ortholog of *A. nidulans* NapA) or Hsf1 (heat shock factor 1) before it binds to target promoters in response to oxidative stress and/or heat shock [37]. He and Fassler reported that Skn7 binds to the oxidative stress response element (OSRE) (5'GGCNNGGC3', 5'GGCNGGC3', 5'GGCNAGA3', or 5'GGCNNAGA3') in the promoters of *CTT1* (cytosolic catalase), *CCP1* (mytochondrial cytochrome c peroxidase), *TSA1* (cytosolic thioredoxin peroxidase), *AHP1* (alkyl hydroperoxide reductase) as target genes and induces their expression in response to H_2_O_2_. Morgan and co-workers reported that Skn7 binds to the sequence 5'CCGAAA3' in *TRX2* (thioredoxin) promoter [34,79]. Skn7 binds to the heat shock element (HSE) in heat shock genes by cooperation with Hsf1 especially in response to hydrogen peroxide [74]. It seems that Skn7 binds to the promoters of the specific target genes by interaction with different partner proteins in response to different stress conditions. 

Similar to Skn7, Pap1 and Prr1 (*S. pombe* orthologs of Yap1 and Skn7) are required for activation of the *ctt1* and *trr1* in *S. pombe* [80]. 

Interaction of Skn7 with AP-1 orthologs implies an involvement of Skn7, although indirect, in regulation of secondary metabolism. Indeed, Hong and co-workers found a conserved 5-base motif 5'AAGCC3', a recognition site for SrrA, in promoters of the antioxidant genes *cat1* and Mn *sod*, as well as in the promoters of aflatoxin biosynthetic genes, *fas-1* and *ver-1* in *A. parasiticus* [5]. Location of this site in close proximity to ATG translation initiation codon and to AP-1 binding sites in the promoter suggests the physical interaction between SrrA and AP-1, and their involvement in regulation of aflatoxin gene expression.

*A. nidulans* SskA is one of several response regulators in a signal transduction pathway that mediates cellular response to environmental stimuli [76,77]. Despite not being a transcription factor, disruption of *sskA* in *A. nidulans* showed not only a similar phenotype to *srrA* deletion mutants (hypersensitivity to hydrogen peroxide and decreased levels of expression of *catB*) but also hypersensitivity of conidia to hydrogen peroxide and reduced germination efficiency [76]. In contrast to observations by Hagiwara and co-workers, Vargas-Perez and co-workers reported that *sskA* knock-out mutants of *A. nidulans* do not show sensitivity to hydrogen peroxide and that *sskA* is not required for expression of *catB* [77]*.* These observations pointed out at two alternative signaling routes, which function simultaneously and/or in an alternate fashion. Because SrrA is an upstream component in a redox signaling pathway, there is the possible connection for this protein to secondary metabolism.

### 4.3. Pcr1

Pcr1, a bZIP transcription factor, was shown to bind to CRE sites and regulated sexual development by cAMP-PKA in *S. pombe* [42]. Watanabe and co-workers suggested that Pcr1 may form a heterodimer with another bZIP transcription factor Atf1 in *S. pombe* [42]. Pcr1 was required for full response to oxidative stress or heat shock in liquid culture [43]. Similar to Atf1, Pcr1 was required for transcription of most of stress-dependent genes such as *gpx1* (glutathione peroxidase) and *ctt1* (cytosolic catalase). Sanso and co-workers demonstrated that Pcr1 is a phosphoprotein and its dephosphorylation occurs in response to oxidative stress in a Sty1-dependent manner [43].

### 4.4. Myb

Myb transcription factors are conserved in animals, plants, algae, and fungi. Myb proteins are often encoded by multiple genes in an individual organism, possess imperfect helix-turn-helix repeats, and are among a group of transcriptional regulators involved in eukaryotic cell cycle progression; c-m*yb* is an oncogene in mammals [81]. In the plant *Arabidopsis thaliana*, Myb transcription factors are involved in phenylpropanoid and flavonoid biosynthesis [82,83,84] and in a number of responses to different stressors including phosphorous, salinity, cold, dehydration, and osmotic shock [81]. 

An *A. nidulans* Myb transcription factor FlbD is a specific regulator of asexual and sexual development; regulation of sexual development by FlbD is *veA*-independent [85,86]. Myb is also able to perceive cellular red-ox state [86]. In the plant pathogen *Phytophthora sojae*, the Myb transcription factor PsMYB1 is required for zoospore development and zoospore-mediated plant infection [87]. In addition, it has been shown that activation of the SAPK/MAPK pathway directly or indirectly increases transcript levels of PsMYB1 [87].

Given the established stress response involvement of Myb transcription factors, their connection with SAPK/MAPK pathway, and involvement in regulation of secondary metabolism in plants and development in fungi, it is reasonable to anticipate that uncharacterized yet Myb transcription factors may coordinate secondary metabolism and oxidative stress response. 

## 5. Conclusions and Future Research

(1) Signaling pathways utilized to sense oxidative stress and to regulate target gene expression are evolutionary well conserved between filamentous fungi and yeast. Yet, there are particular significant differences between species with regard to their response to damaging levels of ROS. Findings from our laboratory and others indicate that in filamentous fungi, in contrast to yeast, cellular response to excessive ROS incorporates secondary metabolism. Of special importance, a network of oxidative stress-related transcription factors helps coordinate the expression of many downstream target genes including antioxidant genes and genes involved in secondary metabolism. The network transmits signals that arise from a variety of sources through different signaling routes, thus showing a high degree of cross-talk. 

(2) Individual transcription factors fulfill distinct but often overlapping signaling functions. For instance, Yap-1, Nap-1, and APyap1 - all belong to the AP-1 family of transcription factors. These transcription factors show overlapping functions related to ROS signaling in yeast and filamentous fungi and activation of antioxidant target genes. On the other hand, in filamentous fungi these factors perform an additional unique task, which is co-regulation of secondary metabolism. What structural/functional features provide them with this uniqueness? One common characteristic may be the ability to interact with the variety of other transcription factors. 

(3) The connection between ROS, secondary metabolism, and fungal development is a well-known phenomenon but was out of the scope in this review. Nevertheless, it is likely that the same collection of transcription factors discussed above orchestrates regulation of developmental processes during response to oxidative stress. The network may include recently described transcription factors NsdC and NsdD [88]. These zinc-finger transcription factors have been shown to participate in regulation of asexual development and aflatoxin biosynthesis in *A. flavus*, although their involvement in oxidative stress response is currently unclear. Research efforts focused to explore regulatory ties between ROS, secondary metabolism, and fungal development help us to view secondary metabolism interconnected with essential processes in the fungal cell.

(4) Despite strong evidence for a close regulatory connection between secondary metabolism and antioxidant defense in response to elevated ROS levels, a question arises: “Could secondary metabolism be regulated independently from the oxidative stress”? Deletion or overexpression of RsmA, a bZIP transcription factor, which is a Yap3 ortholog in *A. nidulans*, has been shown to specifically affect sterigmatocystin biosynthesis with no effect on tolerance/sensitivity to ROS under investigated conditions [27,89]. Therefore, it is likely that such pathway(s) exist, but this remains an interesting and still open question. 

(5) A better understanding of the transcriptional regulatory network that coordinates secondary metabolism and stress response will provide new insights into secondary metabolism in fungi and may help us to develop stress tolerant industrial fungal strains for beneficial secondary metabolite production and to find new targets for eliminating detrimental secondary metabolite production from fungi. 
